# Functional physiological phenotyping and transcriptome analysis provide new insight into strawberry growth and water consumption

**DOI:** 10.3389/fpls.2022.1074132

**Published:** 2022-11-24

**Authors:** Lili Jiang, Ting Sun, Xiaofang Wang, Xiaojuan Zong, Chong Wu

**Affiliations:** ^1^ Shandong Institute of Pomology, Taian, Shandong, China; ^2^ Key Laboratory of Specialty Agri-product Quality and Hazard Controlling Technology of Zhejiang Province, College of Life Sciences, China Jiliang University, Hangzhou, China

**Keywords:** strawberry, irrigation, critical soil water content, balance, growth, phenotyping

## Abstract

Global warming is expected to increase agricultural water scarcity; thus, optimized irrigation schedules are important and timely for sustainable crop production. Deficit irrigation, which balances crop growth and water consumption, has been proposed, but the critical threshold is not easily quantified. Here, we conducted experiments on strawberry plants subjecting progressive drought following various water recovery treatments on the high-throughput physiological phenotyping system “Plantarray”. The critical soil water contents (θ_cri_), below which the plant transpiration significantly decreased, were calculated from the inflection point of the transpiration rate (Tr) - volumetric soil water content (VWC) curve fitted by a piecewise function. The physiological traits of water relations were compared between the well-watered plants (CK), plants subjecting the treatment of rewatering at the point of θ_cri_ following progressive drought (WR_θ_cri_), and the plants subjecting the treatment of rewatering at severe drought following progressive drought (WR_SD). The results showed that midday Tr, daily transpiration (E), and biomass gain of the plants under WR_θ_cri_ treatment were equivalent to CK during the whole course of the experiment, but those under WR_SD treatment were significantly lower than CK during the water stress phase that could not recover even after rehydration. To explore the gene regulatory mechanisms, transcriptome analysis of the samples collected 12 h before, 12 h post and 36 h post water recovery in the three treatments was conducted. GO and KEGG enrichment analyses for the differentially expressed genes indicated that genes involved in mineral absorption and flavonoid biosynthesis were among the most striking transcriptionally reversible genes under the WR_θ_cri_ treatment. Functional physiological phenotyping and transcriptome data provide new insight into a potential, quantitative, and balanceable water-saving strategy for strawberry irrigation and other agricultural crops.

## 1 Introduction

Human-induced global warming is presently increasing, which increases the odds of worsening drought (Falkland and White, 2020). The total land area subject to drought will increase, and agricultural droughts will become more frequent and severe due to the increasing evapotranspiration associated with enhanced temperature, decreased relative humidity and increasing net radiation under global warming (IPCC, 2021). In the summer of recent years, La Niña typically leads to warm temperatures and accompanying drought across the Northern Hemisphere, which increases the likelihood of severe impacts on agriculture and ecosystems (Cates et al., 2022). Agricultural water use for irrigation accounts for 70% of the total water consumption worldwide, followed by industrial and municipal use (Chen et al., 2018); therefore, optimizing agricultural water use is of great significance for alleviating water scarcity.

Strawberry (*Fragaria × annanasa* Duch.) is one of the most economically important horticultural crops in the world. Shallow rooting systems, high leaf area and large fruit result in strawberry being a water-intensive crop (Ali Sarıdaş et al., 2021). Consumptive water use of strawberry has been reported to be between ~300 and ~800 mm (Lozano et al., 2016). As a protected cultivation fruit, strawberry is generally grown under plastic tunnels during the entire season (Galati et al., 2020); thus, intensive use of water will significantly reduce planting costs and save water resources. To date, most irrigation scheduling is intuitive or qualitative, which generally leads to overirrigation (Kapur et al., 2018). The irrigation scheduling recommended by the FAO depends on the soil water balance with crop water demand, i.e., reference crop evapotranspiration (ET_c_) calculated by the Penman–Monteith equation (Steduto et al., 2012). This approach was recently applied to strawberry in southern Spain, which saved water by ~40% (Gavilán et al., 2021). Nevertheless, prolonged soil saturation under water balance is not entirely satisfactory because of various root diseases and nutrient leaching (Villa et al., 2022). Deficit irrigation, an irrigation practice in which a crop is watered below full crop water demand, is reported to significantly increase fruit quality, such as sugars (Zarrouk et al., 2015), without compromising production (Mirás-Avalos et al., 2016; Permanhani et al., 2016). Rodríguez Pleguezuelo et al. (2018) set the deficit irrigation condition to 33%, 50%, and 75% of the ET_c_ and found that 50% deficit irrigation obtained the highest mango yield. Williams et al. (2009) studied the response of vines to different irrigation inputs between 20 and 140% of the ET_c_ in a California environment and found that maximum yields were achieved at 60–80% of the ET_c_. In a case study of strawberry irrigation regimes in a Mediterranean environment, the maximum total berry yield was attained at 75% of the ET_c_, while there was a significant decrease at 50% and no benefit at 100% and 125% of the ET_c_ (Kapur et al., 2018). Deficit irrigation is reported as an effective technology to improve water use efficiency and reduce water waste on the premise of maintaining the quality of agricultural products, especially in the horticultural crops represented by fruits and vegetables (Yang et al., 2022); however, by far, the threshold of water deficit balancing plant growth and water saving is still expertise-based when scheduling water application.

With the rapid development of phenomics in recent years, functional physiological traits, such as diurnal transpiration (Tr), could be nondestructively monitored with a high-throughput method (Halperin et al., 2017; Li et al., 2020; Gosa et al., 2022). Plant water use behavior is then detected *via* the dynamic response of transpiration to a progressively decreasing soil water content (SWC) (Vadez et al., 2014; Halperin et al., 2017). It has widely been observed that there was no significant difference in whole-plant transpiration rates during the initial days of irrigation reduction, and stomatal conductance (g_s_) is limited and transpiration significantly decreased only under lower SWCs (Halperin et al., 2017; Luo et al., 2022). The response curves of transpiration against water stress are generally described as a piecewise function in crop models (e.g., AquaCrop) or physiological studies of water response (Steduto et al., 2012; Vadez et al., 2014), where Tr decreased linearly after SWC exceeded a critical threshold (θ_cri_). θ_cri_ is a genotype-dependent parameter that indicates conservative and profligate water use strategies for drought tolerance (Casadebaig et al., 2008; Luo et al., 2022). Based on high-throughput lysimeter systems, genotypic differences in θ_cri_ in different populations have been detected and used for genome-wide association studies (GWAS) (Xu et al., 2015; Wu et al., 2021). Water deficit reaches a tipping point, below which crop growth will be significantly influenced by stomatal closure; hence, genotype-dependent θ_cri_ could be a quantifiable threshold for deficit irrigation. Nevertheless, to date, θ_cri_ has rarely been applied to irrigation practices as a threshold. To test this hypothesis, we conducted progressive drought experiments with three genotypes of strawberry, which has different drought tolerances. The physiological parameters obtained by the high-throughput physiological phenotypic platform were compared for two sets of progressive drought experiments where water recovery was conducted at θ_cri_ and severe water deficit. RNA-seq experiments were also conducted before and after water recovery to gain insights into the gene regulatory profiles under the respective conditions, and thus underlying mechanism governing the association between soil water content on growth and water consumption of strawberry will be revealed. Our study provided a potential, quantitative, and balanceable water-saving strategy for strawberry irrigation and other agricultural crops.

## 2 Materials and methods

### 2.1 Plant materials, cultivation and management

**Figure 1:**
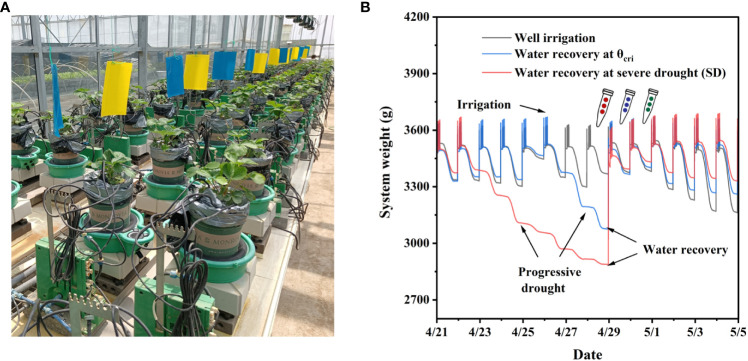
Strawberry experiments conducted with the high-throughput phenotyping platform “Plantarray”. **(A)**, Overview of the automated physiological phenotyping array loaded with strawberry seedlings. **(B)**, System weights that were recorded every 3 minutes during the experimental course. Three irrigation regimes are scheduling as well irrigation across the whole period (CK), water recovery at the critical threshold (θ_cri_) when Tr starts to reduce significantly (WR_θ_cri_), and water recovery at severe drought (WR_SD). The WR_θ_cri_ and WR_SD experiments were divided into the well-irrigated, progressive drought, and water recovery phases.

Three varieties of strawberry, i.e., Xiangye, Hongyan, and ZhangJi, introduced from Japan and widely cultivated in China were used in this study, which differ in drought-tolerance according to the preliminary screening by field survey. The experiments were carried out in a semi-controlled greenhouse (L×W×H: 10 m×5 m×4 m) in January and April of 2022 in Huai’an (119°01′E, 33°35′N), Jiangsu Province, China. One seedling at the four-leaf stage was transplanted per pot (upper diameter 16 cm, lower diameter 13 cm, height 18 cm, 1.5 L in total) with profile porous ceramic substrate (PPC, diameter: 0.2 mm, pH: 5.5 ± 1, porosity: 74, and CEC: 33.6 mEq/100 g). Seven replicates (three for destructive sampling) for each genotype were set in a completely randomized design on the high-throughput physiological phenotyping platform, also known as ‘Plantarray’ (**Figure 1A**, Plant-Ditech, Israel, Halperin et al., 2017). Water and nutrient supplements (Yamazaki nutrient solution) were controlled by the automatic irrigation system of “Plantarray” (Xu et al., 2015).

### 2.2 Experimental design

As shown in **Figure 1B**, the irrigation regimes were scheduled as sufficient irrigation across the whole period (CK), water recovery following progressive drought at the critical threshold (θ_cri_) when Tr starts to reduce significantly (WR_θ_cri_), and water recovery following progressive drought at severe drought (WR_SD), where the wilting leaves were observed. Experiments of WR_θ_cri_ and WR_SD included three phases, i.e., sufficient irrigation, progressive drought and water recovery. The water supply of the WR_SD treatment was stopped 5 days earlier than WR_θ_cri_ (according to the preliminary experiment) to guarantee the same day of water recovery; thus, the functional physiological traits of different treatments could be compared under the same environmental conditions. At the sufficient irrigation and water recovery phases, nutrient solution was provided by irrigation for 240 seconds (oversaturated) at 0:00, 1:00, 2:00, and 3:00 hours, and no nutrient solution was supplied during the progressive drought phase.

### 2.3 Meteorological and functional physiological parameter acquisition

**Figure 2:**
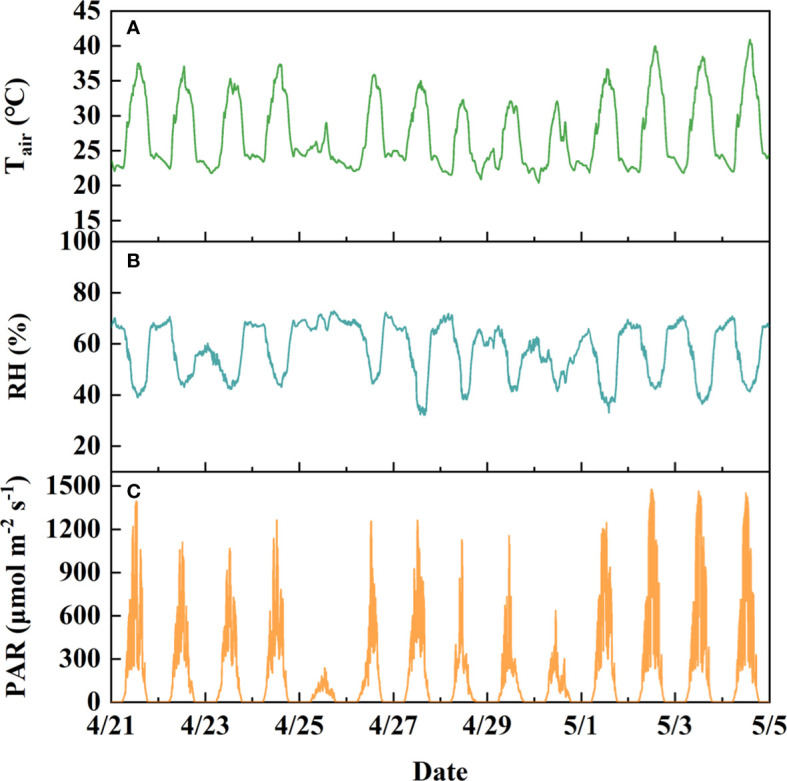
Parameters of the soil–plant–atmosphere continuum monitored by “Plantarray”. **(A)**, air temperature (T_air_, °C). **(B)**, Relative humidity (RH, %). **(C)**, Photosynthetic active radiation (PAR, μmol m^-2^ s^-1^). The data shown in the graph were obtained from the spring-season experiment in 2022.

Air temperature (T_air_, °C) and relative humidity (RH, %), photosynthetically active radiation (PAR, μmolm^−2^ s^−1^) above the canopy, and soil volumetric water content (VWC, m^3^ m^−3^) in each pot were measured by the VP-4 sensor (Decagon Devices, Pullman, Wash, USA), 5TM sensor (Decagon Devices, Pullman, Wash, USA) and PYR solar radiation sensor (Decagon Devices, Pullman, Wash, USA), respectively. Diurnal variations in T_air_, RH and PAR during the spring growth season are exhibited in **Figure 2**. The vapor pressure deficit (VPD, kPa) was calculated according to Halperin et al. (2017).

The physiological parameters for plant water relations were obtained from the “Plantarray” system. This system provides a simultaneous measurement and detailed physiological response profile for plants in each pot in the array over time periods ranging from a few minutes to the entire growing season under normal, stress and recovery conditions and at any phenological stage (Li et al., 2020). The whole-plant transpiration rates (Tr, g min^-1^), daily whole-plant transpiration (E, g d^-1^), and daily fresh plant growth (PG, g d^-1^, only under overirrigated conditions) were calculated using the SPAC software implemented in the “Plantarray” system according to the continuously monitored system weight. Briefly, Tr was computed by multiplying the first derivative of the measured system weight by -1. E was calculated by the difference in the measured system weight at predawn (averaged between 4:00 and 4:30) and that at evening (averaged between 19:00 and 19:30). During the well irrigation phase, the PG was calculated as the difference in the predawn system weights on two adjacent days. Difference between measurement data were compared through analysis of variance and that between groups with Q test.

### 2.4 Critical soil water content (θ_cri_) determined by the dynamic water response curve

Daily whole-plant midday transpiration (Tr_m_, averaged over the period between 12:00 and 14:00) was fitted to a two-piecewise linear function of the corresponding soil volumetric water content (VWC) during the dynamic period of water deficit, which was described in our previous study (Wu et al., 2021). To offset the influence of daily environmental variation, Tr_m_ is normalized to VPD (Tr_m,VPD_). The dynamic water response function is then determined using the following equation:


(1) $${\text{Tr}}_{\text{m},\text{VPD}}=\{\begin{array}{ll} a, & \text{VWC}\geq {\text{θ}}_{\text{cri}}\\ a+k\times (\text{VWC}-{\text{θ}}_{\text{cri}}), & \text{VWC}<{\text{θ}}_{\text{cri}} \end{array}$$

where θ_cri_ is the critical soil water content quantified by the VWC in this study, a is the maximum Tr_m,VPD_, *k* is the decreasing slope of Tr_m,VPD_ when VWC is below θ_cri_.

### 2.5 Sampling and RNA isolation and RNA-seq

New fully expanded leaves of strawberry variety Xiangye of CK, WR_θ_cri_ and WR_SD treatments were sampled at approximately 12:00 am the day before water recovery around the end of progressive drought (12 h before water recovery, i.e., CK 12 h-, WR_θ_cri_ 12 h-, WR_SD 12 h-), the day of water recovery (12 h after water recovery, i.e., CK 12 h+, WR_θ_cri_ 12 h+, WR_SD 12 h+), and the day after water recovery (36 h after water recovery, i.e., CK 36 h+, WR_θ_cri_ 36 h+, WR_SD 36 h+), respectively. Three biological replicates (pots) were set for each treatment. Total RNA was extracted using TRIzol reagent (Invitrogen, Carlsbad, CA). Five micrograms of total RNA from each sample was used to construct an RNA-Seq library by using the TruSeq RNA Sample Preparation Kit according to the manufacturer’s instructions (Illumina, San Diego, CA). A total of 27 libraries were constructed and then sequenced on the Novaseq 6000 platform. The accession number for the sequence data is PRJNA891140 (https://www.ncbi.nlm.nih.gov/search/all/?term=PRJNA891140).

### 2.6 Raw data processing, pairwise differential expression analysis and functional analysis

The raw reads were filtered and trimmed using SeqPrep (https://github.com/jstjohn/SeqPrep) and Sickle (https://github.com/najoshi/sickle) with default parameters. The clean reads were combined and aligned to the strawberry reference genomes (https://www.rosaceae.org/Analysis/9642085). The count of the mapped reads from each sample was derived and normalized to fragments per kilobase of transcript length per million mapped reads (FPKM) for each predicted transcript using Cufflinks (v2.2.1).

Pairwise comparisons were made between samples collected at the same timepoint and different phases. To reduce noise and false results, only genes having an FPKM ≥ 1 in at least 3 of the 6 samples of a comparison (3 replicates each sample) and a coefficient of variation (CV)< 0.2 in the 3 replicates were considered. The genes exhibiting a difference of at least twofold change with the corrected *P* value after adjusting with false discovery rate (FDR) ≤ 0.05 were considered significantly differentially expressed.

Gene functional categories (GO enrichments) were analyzed using AgriGO V2.0 (Tian et al., 2017) under a *q*-value threshold of 0.01 for statistical significance. Unigenes were annotated to corresponding KEGG gene/KEGG ortholog descriptions and their associated pathways using the KEGG Automatic Annotation Server (KAAS, http://www.genome.jp/tools/kaas/) (Moriya et al., 2007). The list of gene identifiers and log2FC values from each treatment were imported into MapMan software version 3.5.1R2 and assigned to functional categories using the strawberry mapping file obtained by Mercator 4.

## 3 Results

### 3.1 Dynamic patterns of Tr in response to soil water contents decline

**Figure 3:**
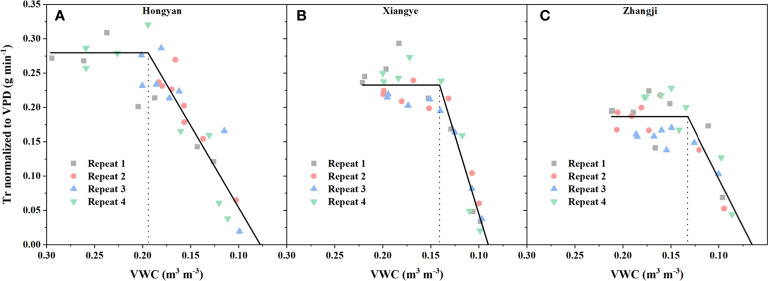
Midday Tr normalized to VPD (Tr_m,VPD_) against soil water content (VWC) for different strawberry varieties. The Tr_m,VPD_ and VWC data were both averaged between 12:00 and 14:00 and then fitted with a piecewise function (Eq. 1). The inflection point of the fitting curve was determined as the critical soil water content, i.e., θ_cri_, below which Tr_m,VPD_ decreased linearly. Three strawberry varieties, i.e., Hongyan **(A)**, Xiangye **(B)** and Zhangji **(C)**, were used in the experiment.

The midday Tr normalized to VPD (Tr_m,VPD_) and soil VWC averaged between 12:00 and 14:00 were well fitted with a piecewise function (**Figure 3**, R^2^ ranged from 0.82 to 0.91 for the dataset obtained from spring season). The transpiration variation caused by environmental fluctuations, as shown in **Figure 2**, was largely smoothed by dividing by VPD, where Tr_m,VPD_ remained relatively stable when there was no severe water deficit (**Figure 3**). The inflection point of the fitting curve was determined as the critical soil water content, i.e., θ_cri_ in Eq. 1, below which Tr_m,VPD_ decreased linearly. θ_cri_, which represents the sensitivity of stomatal closure (Halperin et al., 2017), varied within different varieties of strawberry. The θ_cri_ of Hongyan was highest (0.1944 ± 0.0087, **Figure 3A**), followed by Xiangye (0.1410 ± 0.0031, **Figure 3B**), and Zhangji (0.1327 ± 0.0087, **Figure 3C**), indicating that within the three strawberry varieties, Hongyan was most sensitive to water deficit and reduced Tr the earliest to survive under drought. In addition, the descending slope, i.e., k in Eq. 1 also differed within varieties, where Xiangye (4.6011 ± 0.4396, **Figure 3B**) was significantly different from the other two (Hongyan: 2.3915 ± 0.3178, **Figure 3C**; Zhangji: 2.7851 ± 0.7534, **Figure 3A**), suggesting a faster process of stomatal closure. There was no significant difference between θ_cri_ of a particular variety obtained from the winter season and spring season, but there was little difference for k. That is, θ_cri_is a relatively conservative, genotype-independent parameter that characterizes the dynamic pattern of Tr in response to the soil water content.

### 3.2 Physiological traits before and after water recovery

**Figure 4:**
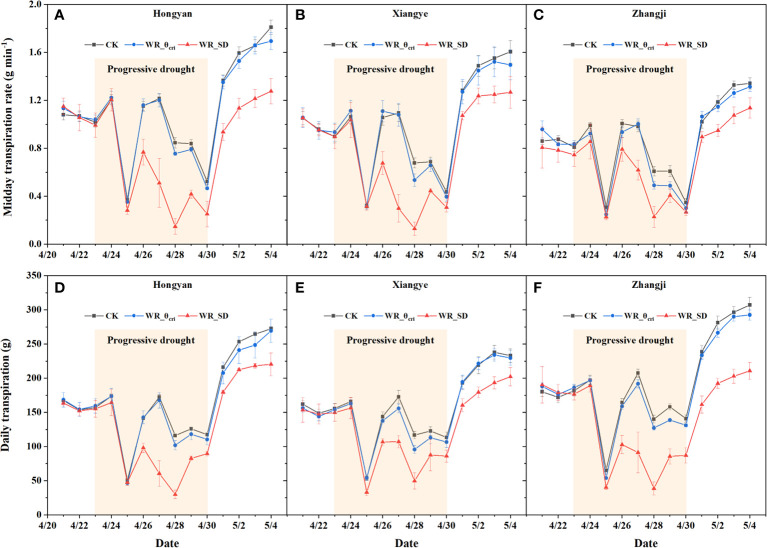
Midday transpiration rate (Tr_m_) and daily transpiration (E) of different treatments during the spring season for strawberry varieties. Three treatments, i.e., well irrigation across the whole period (CK), water recovery at the critical threshold (θ_cri_) when Tr started to reduce significantly (WR_θ_cri_), and water recovery under severe drought (WR_SD), are shown in the graph. Three strawberry varieties, i.e., Hongyan **(A, D)**, Xiangye **(B, E)** and Zhangji **(C, F)**, were used in the experiment.

The midday Tr (Tr_m_) and daily transpiration (E) of treatments with three irrigation schedules, i.e., well irrigation (CK), water recovery at θ_cri_ (WR_θ_cri_), and water recovery under severe drought (WR_SD), were compared, as shown in **Figure 4** for Hongyan, Xiangye, and Zhangji. There was no significant difference in Tr_m_ or E during the period before progressive drought treatment within all three varieties (**Figure 4**). With the experiment, WR_θ_cri_ was close to or slightly slower than CK (no significant difference in two-way ANOVA), where Tr_m_ and E were both reduced by ~6% averaged for three varieties across the progressive drought phase (blue line in shaded area of **Figure 4**). However, the Tr_m_ and E of WR_SD both decreased significantly compared to CK (*P<*0.01), by ~37% and ~35%, respectively (red line in shaded area of **Figure 4**). Within the three varieties, the Tr_m_ of Hongyan, which is highly sensitive to water deficit according to θ_cri_, decreased the most during the progressive drought period in the WR_SD treatment (~42%, **Figure 4A**), followed by Xiangye (~37%, **Figure 4B**) and Zhangji (~31%, **Figure 4C**), but was different for E (Zhangji: reduced by ~41%, **Figure 4D**; Hongyan: reduced by ~34%, **Figure 4E**; Xiangye: reduced by ~31%, **Figure 4F**). After water recovery, the Tr_m_ and E of WR_θ_cri_ recovered rapidly to levels similar to those of CK, where Tr_m_ and E only decreased by ~2% averaged for the three varieties across the water recovery phase (**Figure 4**). In accordance with Tr_m_ and E during the progressive drought phase, the recovery of Tr_m_ was poorest for Hongyan (reduced by ~29%, **Figure 4A**), followed by Xiangye (~18%, **Figure 4B**) and Zhangji (~16%, **Figure 4C**), while there was the largest gap between the E values of WR_SD and CK for Zhangji (reduced by ~32%, **Figure 4F**), followed by Hongyan (~17%, **Figure 4D**) and Xiangye (~16%, **Figure 4E**).

Daily plant growth (fresh weight, g) was also detected nondestructively by the “Plantarray” system during the water recovery phase, and water use efficiency (WUE) during the 5-day window after water recovery was also calculated by accumulated plant growth and E. The daily plant growth of WR_SD during the water recovery phase was significantly lower than WR_θ_cri_ and CK in all three varieties, mainly due to the daily transpiration, as there was no significant difference in WUE either between varieties or treatment (**Table 1**).

**Table 1:** Daily Plant growth and WUE during the period after water recovery.

Variety		Xiangye			Hongyan			Zhangji		
Treatment		CK	WR_θ_cri_	WR_SD	CK	WR_θ_cri_	WR_SD	CK	WR_θ_cri_	WR_SD
Plant growth (g)	4/30	5.47 ± 0.58	5.22 ± 0.35	4.16 ± 0.39	5.91 ± 0.35	5.68 ± 0.17	3.99 ± 0.81	5.6 ± 0.38	4.51 ± 0.34	4.15 ± 0.74
	5/1	9.36 ± 0.97	9.56 ± 1.08	7.94 ± 0.89	10.99 ± 0.68	10.13 ± 0.81	7.14 ± 0.85	8.24 ± 0.58	9.16 ± 0.44	7.04 ± 0.97
	5/2	11.33 ± 1.14	11.1 ± 1.22	9.61 ± 0.29	13.2 ± 0.92	12.95 ± 0.91	7.88 ± 0.6	9.95 ± 0.88	10.6 ± 0.5	8.36 ± 1.87
	5/3	11.89 ± 1.04	10.9 ± 1.13	9.88 ± 1.02	13.02 ± 0.84	13.35 ± 0.13	9.18 ± 1.13	11.21 ± 0.65	11.5 ± 0.27	8.18 ± 0.79
	5/4	11.89 ± 1.08	12.05 ± 0.83	9.39 ± 0.83	13.3 ± 0.41	13.07 ± 0.64	9.81 ± 1.35	10.53 ± 0.74	10.98 ± 0.43	8.41 ± 1.55
WUE (g g-1)	5 day	0.044 ± 0.003	0.045 ± 0.003	0.044 ± 0.001	0.045 ± 0.004	0.044 ± 0.001	0.044 ± 0.002	0.045 ± 0.002	0.048 ± 0.003	0.043 ± 0.001

### 3.3 Overview of differentially expressed genes before and after water recovery

**Figure 5:**
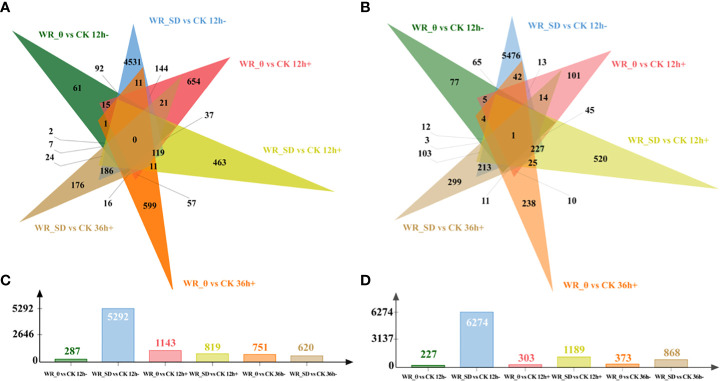
Overview of DEGs before and after water recovery. a-b, Venn diagrams for upregulated **(A)** and downregulated **(B)** DEGs of pairwise comparisons within the entire WR_θ_cri_ vs. CK set and WR_SD vs. CK. c-d, Number of upregulated **(C)** and downregulated **(D)** DEGs of pairwise comparisons within the entire WR_θ_cri_ vs. CK set and WR_SD vs. CK. “CK 12 h-, WR_θ_cri_ 12 h-, WR_SD 12 h-”, “CK 12 h+, WR_θ_cri_ 12 h+, WR_SD 12 h+”, and “CK 36 h+, WR_θ_cri_ 36 h+, WR_SD 36 h+” indicate samples obtained at 12 h before, 12 h after and 36 h after water recovery in the CK, WR_θ_cri_, and WR_SD treatments, respectively.

Physiological traits before and after water recovery indicated that there is an essential difference for strawberry growth between WR_θ_cri_ and WR_SD. To gain insights into the gene regulatory profiles under the respective conditions, gene expressions 12 h before (end of progressive drought, 12 h-), 12 h after (12 h+), and 36 h after (36 h+) water recovery in the WR_θ_cri_ and WR_SD treatments were compared with CK based on RNA-Seq data. An overview of pairwise comparisons is shown in **Figure 5**. The number of both upregulated and downregulated DEGs of WR_SD at 12 h before water recovery compared to CK (WR_SD vs. CK 12 h-) were greatest, while the opposite was true for WR_θ vs. CK 12 h-.

### 3.4 Functional analysis of DEGs identified from different comparisons

#### 3.4.1 Gene ontology enrichment analysis

**Figure 6:**
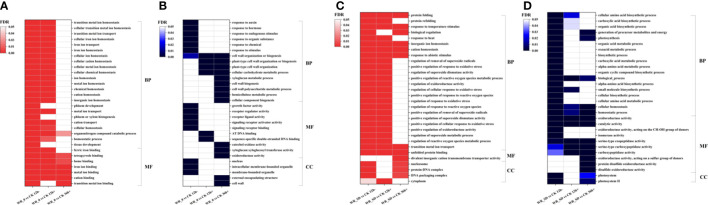
GO terms enriched in DEGs of pairwise comparisons within the entire WR_θ_cri_ vs. CK set and WR_SD vs. CK set. **(A)**, GO terms enriched in upregulated DEGs within the WR_θ_cri_ vs. CK set. **(B)**, GO terms enriched in downregulated DEGs within the WR_θ_cri_ vs. CK set. **(C)**, GO terms enriched in upregulated DEGs within the WR_SD vs. CK set. **(D)**, GO terms enriched in downregulated DEGs within the WR_SD vs. CK set. The top 30 significant (*P*<0.01) GO terms are shown in the graph, and the FDR value of each item was used to draw a heatmap.

We performed GO enrichment analysis on the entire set of DEGs upregulated and downregulated by WR_θ and WR_SD compared to CK at different time points (12 h-, 12 h+ and 36 h+). The top 30 enriched GO terms were selected from each set (FDR<0.05, **Figure 6**). Interestingly, over two-thirds of the top 30 GO terms related to iron homeostasis and transport were enriched in upregulated DEGs of WR_θ vs. CK 12 h- (**Figure 6A**). After water recovery, the abovementioned GO terms were enriched in the upregulated DEGs of WR_θ vs. CK 12 h+ but not significantly enriched in WR_θ vs. CK 36 h+, except for the molecular functions ‘heme binding’, ‘iron ion binding’, ‘metal ion binding’, ‘cation binding’, and ‘transition metal ion binding’ (**Figure 6A**). In addition, GO enrichment analysis of upregulated DEGs of WR_θ vs. CK 12 h- showed enrichment for the biological processes ‘phloem development’, ‘phloem or xylem histogenesis’, and ‘tissue development’ but could not be retrieved for that of WR_θ vs. CK 12 h+ or WR_θ vs. CK 36 h+ (**Figure 6A**).

Stimulus response-related GO terms (e.g., biological processes ‘response to auxin’, ‘response to hormone’, ‘response to endogenous stimulus’, ‘response to organic substance’, ‘response to chemical’, and ‘response to stimulus’; molecular functions ‘receptor regulator activity’, ‘receptor ligand activity’, ‘signaling receptor activator activity’, and ‘signaling receptor binding’) and cell growth-related GO terms (e.g., biological processes ‘cell wall organization or biogenesis’; molecular functions ‘growth factor activity’; and cellular component ‘intracellular membrane-bounded organelle’ and ‘nucleus’) were enriched in downregulated DEGs of WR_θ vs. CK 12 h- but not significantly in WR_θ vs. CK 12 h+ and WR_θ vs. CK 36 h+ (**Figure 6B**). In contrast, cell wall-related GO terms (e.g., biological processes ‘xyloglucan metabolic process’, ‘cell wall biogenesis’, ‘cell wall polysaccharide metabolic process’, ‘cellular component biogenesis; molecular functions ‘xyloglucan: xyloglucosyl transferase activity’; and cellular component ‘external encapsulating structure’, and ‘cell wall’) were specific for downregulated DEGs of WR_θ vs. CK 36 h+ (**Figure 6B**).

The top 30 GO terms enriched in upregulated DEGs of WR_SD vs. CK 12 h- and WR_SD vs. CK 12 h+ were relevant to reactive oxygen species, e.g., biological processes ‘regulation of removal of superoxide radicals’, ‘positive regulation of response to oxidative stress’, ‘regulation of reactive oxygen species metabolic process’, ‘positive regulation of cellular response to oxidative stress’, ‘positive regulation of oxidoreductase activity’, ‘regulation of superoxide metabolic process’, ‘positive regulation of response to reactive oxygen species’, ‘regulation of cellular response to oxidative stress’, ‘positive regulation of reactive oxygen species metabolic process’, ‘regulation of response to oxidative stress’, ‘regulation of response to reactive oxygen species’, ‘positive regulation of superoxide dismutase activity’, ‘regulation of oxidoreductase activity’, and ‘positive regulation of removal of superoxide radicals’ (**Figure 6C**). These GO terms could not be retrieved for the upregulated DEGs of WR_SD vs. CK 36 h+ (**Figure 6C**). Furthermore, the iron transport-related biological processes ‘inorganic ion homeostasis’, ‘cation homeostasis’, and ‘transition metal ion transport’ were always enriched in upregulated DEGs of samples of all time points within the WR_SD vs. CK set (**Figure 6C**).

Many biological processes related to carbohydrate and amino acid metabolism, such as ‘organic acid metabolic process’, ‘oxoacid metabolic process’, ‘biosynthetic process’, ‘carboxylic acid metabolic process’, ‘alpha-amino acid metabolic process’, ‘organic cyclic compound biosynthetic process’, ‘alpha-amino acid biosynthetic process’, ‘cellular biosynthetic process’, and ‘cellular amino acid metabolic process’, were enriched in downregulated DEGs of WR_SD vs. CK 12 h- and recovered in WR_SD vs. CK 12 h+ and WR_SD vs. CK 36 h+ (**Figure 6D**). However, biological progress ‘cellular homeostasis’, ‘homeostatic process’, and molecular functions ‘oxidoreductase activity’, ‘catalytic activity’, ‘serine-type exopeptidase activity’, ‘serine-type carboxypeptidase activity’, ‘carboxypeptidase activity’ were always enriched in downregulated DEGs of samples of all time points within the WR_SD vs. CK set (**Figure 6D**). Some photosynthesis-related GO terms were enriched in the downregulated DEGs of WR_SD vs. CK 12 h- and recovered in WR_SD vs. CK 12 h+; however, they still exhibited enrichment in WR_SD vs. CK 36 h+ (**Figure 6D**).

#### 3.4.2 KEGG pathway enrichment analysis

**Figure 7:**
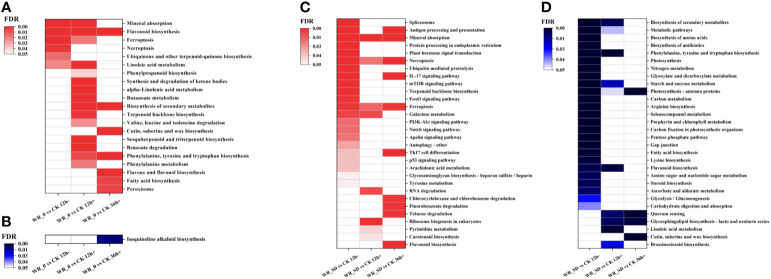
KEGG pathways enriched in DEGs of pairwise comparisons within the entire WR_θ_cri_ vs. CK set and WR_SD vs. CK set. **(A)**, KEGG pathways enriched in upregulated DEGs within the WR_θ_cri_ vs. CK set. **(B)**, KEGG pathways enriched in downregulated DEGs within the WR_θ_cri_ vs. CK set. **(C)**, KEGG pathways enriched in upregulated DEGs within the WR_SD vs. CK set. **(D)**, KEGG pathways enriched in downregulated DEGs within the WR_SD vs. CK set. The top 30 significant (*P*<0.01) KEGG pathways are shown in the graph, and the FDR value of each item was used to draw a heatmap.

To understand the metabolic or signaling pathways involved in the response to drought and water recovery, we mapped upregulated and downregulated DEGs to the Kyoto Encyclopedia of Genes and Genomes (KEGG) database to retrieve the pathways involved in each comparison. In total, 21 and 1 significantly (FDR<0.05) enriched pathways were identified for all upregulated and downregulated DEGs in the WR_θ vs. CK set, respectively (**Figure 7**). Upregulated DEGs of WR_θ vs. CK 12 h- were involved in mineral absorption, flavonoid biosynthesis, ferroptosis, necroptosis, ubiquinone and other terpenoid-quinone biosynthesis, and linoleic acid metabolism. After water recovery, necroptosis, ubiquinone and other terpenoid-quinone biosynthesis were no longer significantly enriched in WR_θ vs. CK 12 h+, while in WR_θ vs. CK 36 h+, mineral absorption, ferroptosis, and linoleic acid metabolism were no longer enriched (**Figure 7A**). Cutin, suberine and wax biosynthesis; flavone and flavonol biosynthesis; fatty acid biosynthesis; and peroxisome biosynthesis were only enriched in WR_θ vs. CK 36 h+. Downregulated DEGs were enriched only in the KEGG pathway of isoquinoline alkaloid biosynthesis in WR_θ vs. CK 36 h+ (**Figure 7B**).

Most upregulated DEGs were significantly enriched in WR_SD vs. CK 12 h- but no longer significant in WR_SD vs. CK 12 h+ and WR_SD vs. CK 36 h+, e.g., spliceosome, protein processing in endoplasmic reticulum, plant hormone signal transduction, ubiquitin-mediated proteolysis, mTOR signaling pathway, and terpenoid backbone biosynthesis (**Figure 7C**). Notably, mineral absorption, necroptosis, and ferroptosis were all enriched in the WR_SD vs. CK set. Moreover, chlorocyclohexane and chlorobenzene degradation, fluorobenzoate degradation, toluene degradation, and flavonoid biosynthesis were enriched only in WR_SD vs. CK36 h+ (**Figure 7C**).

Similarly, most downregulated DEGs were enriched only in WR_SD vs. CK 12 h-, which are nitrogen- or carbon-related pathways, e.g., ‘biosynthesis of amino acids’, ‘photosynthesis’, ‘nitrogen metabolism’, ‘carbon metabolism’, ‘arginine biosynthesis’, ‘carbon fixation in photosynthetic organisms’, ‘pentose phosphate pathway’, ‘lysine biosynthesis’, ‘amino sugar and nucleotide sugar metabolism’, ‘glycolysis/gluconeogenesis’, and ‘carbohydrate digestion and absorption’ (**Figure 7D**). Some secondary metabolism pathways were significant in WR_SD vs. CK 12 h- and WR_SD vs. CK 12 h+, such as ‘flavonoid biosynthesis’. Moreover, ‘cutin, suberine and wax biosynthesis’ was only enriched in WR_SD vs. CK 36 h+ (**Figure 7D**).

#### 3.4.3 DEG visualization for metabolism pathway

**Figure 8:**
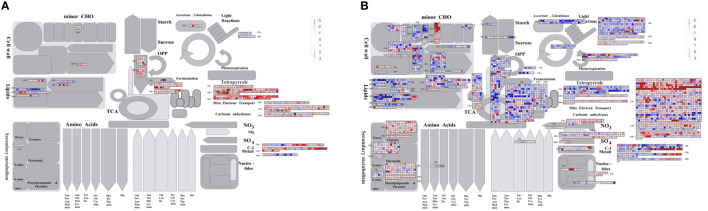
Visualization of DEGs of pairwise comparisons within the entire WR_θ_cri_ vs. CK set **(A)** and WR_SD vs. CK **(B)** set enriched in metabolism pathways. The list of gene identifiers and log2FC values from each treatment were imported into MapMan software version 3.5.1R2 and assigned to functional categories using the strawberry mapping file obtained by Mercator 4.

We then visualized DEGs enriched in metabolic pathways using MapMan, and the metabolism overview of different comparisons is shown in **Figure 8**. For DEGs of the WR_θ vs. CK set, we focused on the transition metal homeostasis and terpenoids category, where large numbers of DEGs were significantly enriched. DEGs related to negative regulators of iron deficiency responses ‘HRZs/BTS’, bHLH-Ib-class transcriptional regulator, and metal cation transporter ‘NRAMP’ were downregulated, while metallothionein ‘MT’, iron storage protein ‘ferritin’, ferric cation-chelator transporter ‘YSL’, and iron transporters ‘VIT’ were upregulated. In contrast, in WR_θ vs. CK 36 h+, DEGs related to metallothionein ‘MT’ were all significantly downregulated, and those of bHLH-Ib-class transcriptional regulators were upregulated. In the tetrapyrrole category, DEGs related to flavonoid biosynthesis, e.g., chalcone synthase (CHS), flavanone 3-hydroxylase (F3H), dihydroflavonol-4-reductase (DFR), anthocyanidin synthase (ANS), leucoanthocyanidin reductase (LAR), and CoA biosynthesis, e.g., cinnamate 4-hydroxylase (C4H), and 4-coumarate-CoA ligase (4CL), were upregulated in WR_θ vs. CK 12 h-. In WR_θ vs. CK 12 h+ and WR_θ vs. CK 36 h+, in addition to the above flavonoid biosynthesis- and CoA biosynthesis-related genes, upregulated DEGs related to the mevalonate pathway, terpene biosynthesis, and cycloartenol biosynthesis were observed.

For DEGs of the WR_θ vs. CK set, DEGs in the protein homeostasis, nitrogen assimilation, transition metal homeostasis, and terpenoid categories were considered. In WR_SD vs. CK 12 h-, genes in the protein homeostasis category were significantly regulated, as a large number of upregulated DEGs were enriched in ER quality control (ERQC) machinery (e.g., membrane-anchored lectin chaperone ‘CNX’, ER luminal lectin chaperone ‘CRT’), mitochondrial Hsp70 chaperone system, Hsp90 chaperone system, Hsp60 chaperone system, N-degron pathways (e.g., GID ubiquitination complex), ubiquitin-fold protein conjugation (e.g., ubiquitin-conjugating enzyme), ATG8-phosphatidylethanolamine conjugation system, cysteine-type peptidase activities (e.g., subclass CTB cysteine protease, subclass SAG12 cysteine protease, subclass RD19 cysteine protease). However, in WR_SD vs. CK 12 h+ and WR_SD vs. CK 36 h+, DEGs enriched in the above pathway were significantly reduced. In the entire WR_SD vs. CK set, in addition to the aforementioned genes enriched in the transition metal homeostasis category of the WR_θ vs. CK set, genes related to the nitrate uptake system (e.g., nitrate transporter ‘NRT1.1’, ‘NRT2’, and ‘NRT3’, signaling factor ‘NRG2’, transcriptional repressor ‘NIGT’, signaling factor ‘NRG2’), ammonium assimilation (e.g., cytosolic glutamine synthetase ‘GLN1’, glutamate dehydrogenase), sulfate uptake system (sulfate transporter ‘SULTR1’, sulfite oxidase, regulatory protein ‘SDI’), and phosphate signaling (transcription factor ‘PHR1’, regulatory protein ‘SPX’, phosphate transporter ‘PHT1’, ‘PHO1’) were enriched. In the terpenoid classification, DEGs of WR_SD vs. CK 12 h- and WR_SD vs. CK 12 h+ were enriched in the mevalonate pathway, methylerythritol phosphate (MEP) pathway, isoprenyl diphosphate biosynthesis, terpene biosynthesis, CoA biosynthesis, and flavonoid biosynthesis, which were mostly downregulated. Similar to WR_SD vs. CK 12 h-, in WR_SD vs. CK 36 h-, DEGs were enriched in the flavonoid biosynthesis pathway, and most of them were upregulated.

## 4 Discussion

### 4.1 The potential of θ_cri_ as the threshold for deficit irrigation

Previous studies have reported that water recovery under moderate drought will not reduce and even increase crop growth and yield, but the threshold of deficit irrigation is unclear or determined empirically according to different ratios of ETc (Williams et al., 2009; Kapur et al., 2018). Our study determined the inflection point (θ_cri_) of the Tr_m,VPD_-VWC curve fitted by a piecewise function as the critical soil water content under drought (**Figure 3**) and explored the potential of θ_cri_ as the threshold of deficit irrigation. Midday Tr, daily E before and after water recovery, and daily plant growth and WUE after water recovery obtained from the “Plantarray” phenotyping system indicated that there was no significant difference in physiological traits between water recovery at θ_cri_ (WR_θ_cri_) and CK, but they were significantly reduced when water recovery occurred under severe drought except WUE (**Figure 4**, **Table 1**). In modeling studies, Tr/VPD is scaled to biomass accumulation (Steduto et al., 2012; Vadez et al., 2014); therefore, θ_cri_ also represents the threshold when water stress significantly affects biomass assimilation. GO and KEGG analyses also supported that photosynthesis-related processes and carbohydrate pathways were downregulated in the WR_SD vs. CK set but not significant in the WR_θ_cri_ vs. CK set (**Figures 6**, **7**). Our study indicated that θ_cri_ was a genotype-independent parameter characterizing dynamic plant water use behavior. With the rapid development of phenomics, high-throughput screening of θ_cri_ has been realized with low cost, thus making it possible to make irrigation schedules individually according to the θ_cri_ of various varieties (Li et al., 2020). As no irrigation was applied during the progressive drought phase of WR_θ_cri_, this method was obviously more water-saving than the ET_c_ methods depending on daily reference evapotranspiration.

### 4.2 Molecular mechanism underlying WR_θ_cri_ distinguished from WR_SD

GO terms and KEGG pathways both indicated that mineral absorption is a vital physiological process influenced by drought, not only in the WR_θ_cri_ vs. CK set but also in WR_SD vs. CK (**Figures 6**, **7**). A series of DEGs visualized in the metabolism overview also included YSL, NRAMP and VIT, which are widely known as Fe transporters. Iron plays an irreplaceable role in alleviating the stress imposed by drought (Tripathi et al., 2018; Rai et al., 2021). Many studies have demonstrated that the application of Fe nutrition to plants under drought conditions enhances tolerance, yield and yield components (Di Caterina et al., 2007; Pourgholam et al., 2013; Mirjahanmardi and Ehsanzadeh, 2016). Recently, Xu et al. (2021) found that iron homeostasis is disrupted by drought stress in sorghum roots using time-series root RNA-Seq data and that loss of a plant phytosiderophore iron transporter impacts microbial community composition, leading to significant increases in the drought-enriched lineage Actinobacteria. Mineral absorption-related genes were upregulated in WR_θ_cri_ vs. CK 12 h- and WR_θ_cri_ vs. CK 12 h+, but entire WR_SD vs. CK set, suggesting that iron homeostasis was repaired in WR_θ_cri_ treatment 36 h after, while not recovered at severe drought treatment even 36 h after water recovery (**Figure 7A, C**). Moreover, necroptosis and ferroptosis, which are related to programmed cell death, were significantly enriched in upregulated DEGs of the entire WR_SD vs. CK set but were not retrieved after water recovery in WR_θ_cri_ treatment, indicating the reversible injury of WR_θ_cri_ treatment (**Figure 7A, C**). This is also why Tr and plant growth were not recovered in the WR_SD treatment (**Figure 4**, **Table 1**).

Drought stress triggering the biosynthesis of secondary metabolites has been widely reported (Yadava et al., 2019). Flavonoids are low-molecular-weight polyphenolic secondary metabolic compounds and play important roles in drought tolerance (Yadava et al., 2019). Ma et al. (2014) found that flavonoid pathway gene expression and the accumulation of flavonoid compounds were closely related to drought tolerance in wheat. Yang et al. (2020) found that *B. chinense* adapts to drought stress through physiological changes and the regulation of flavonoids in different plant tissues. Meng et al. (2021) demonstrated a role for the CcCIPK14-CcCBL1 complex in drought stress tolerance through the regulation of flavonoid biosynthesis in pigeon pea. Li et al. (2021) researched an overly insensitive drought mutant and indicated that flavonoids improve the drought tolerance of maize seedlings by regulating the homeostasis of reactive oxygen species. Flavonoids prevent stomatal closure by reducing the H_2_O_2_ level in guard cells and alleviate drought-induced ROS damage. Interestingly, our results indicated that DEGs related to flavonoid biosynthesis were upregulated in the entire WR_θ vs. CK set and WR_SD vs. CK 36 h+ but downregulated in WR_SD vs. CK 12 h- and WR_SD vs. CK 12 h+. Although it is more common for genes enriched in flavonoid biosynthesis to be upregulated under drought, key enzyme genes in flavonoid biosynthesis, e.g., F3H, 4CL, IFS, and DFR, have also been observed to be downregulated under severe drought (Gu et al., 2020; Yang et al., 2020). We hypothesize that moderate drought triggers flavonoid biosynthesis to defend against the negative effects of water stress but is inhibited under severe drought until 36 h after water recovery. Shojaie et al. (2016) observed that the total flavonoid concentrations in roots and shoots both reached their highest levels under mild drought stress (Ψw = –0.5 MPa) at the end of the drought experiment, while that of severe drought (Ψw = –0.9 MPa) significantly dropped after it peaked in the early stage, which is in accordance with our results.

## 5 Conclusion

The critical soil water content (θ_cri_) was obtained as the inflection point of the Tr_m,VPD_-VWC curve fitted by a piecewise function, depending on the high-throughput physiological phenotyping system. θ_cri_, below which Tr significantly decreased, highly dependent on the varieties of strawberry. The physiological traits of water relations were compared between the well-watered plants (CK), plants subjecting the treatment of rewatering at the point of θ_cri_ following progressive drought (WR_θ_cri_), and the plants subjecting the treatment of rewatering at severe drought following progressive drought (WR_SD). The results showed that the midday Tr and daily E and plant growth of WR_θ_cri_ were slightly lower or close to those of CK (not significant in two-way ANOVA) during the progressive drought and water recovery phases, but those of WR_SD were significantly lower than those of CK during water stress and did not recover after rehydration. Obviously, WR_θ_cri_ is a potential choice for irrigation schedules, which balances crop growth and water consumption. To detect the molecular mechanism underlying progressive drought and water recovery, transcriptome analysis of samples obtained 12 h before, 12 h after and 36 h after water recovery in the WR_θ_cri_, WR_SD, and CK treatments was conducted. GO terms and KEGG pathways indicated that mineral absorption and flavonoid biosynthesis were significantly regulated under drought. Mineral absorption-related genes were upregulated in WR_θ_cri_ vs. CK 12 h- and WR_θ_cri_ vs. CK 12 h+, but entire WR_SD vs. CK set, suggesting that iron homeostasis process was repaired in WR_θ_cri_ treatment 36 h after, while not recovered at severe drought treatment even 36 h after water recovery. Flavonoid biosynthesis was triggered by moderate drought to defend against the negative effects of water stress and remained after water recovery but was inhibited under severe drought until 36 h after water recovery. In summary, genes involved in mineral absorption and flavonoid biosynthesis were among the most striking transcriptionally reversible genes under the WR_θ_cri_ treatment. Therefore, functional physiological phenotyping and transcriptome data indicated that irrigation at the critical soil water content will reduce water consumption without sacrificing plant growth, which provides a potential, quantitative, and balanceable water-saving strategy for strawberry irrigation and other agricultural crops.

## Data availability statement

The original contributions presented in the study are included in the article/supplementary material. Further inquiries can be directed to the corresponding author.

## Author contributions

LJ, TS, and CW contributed to conception and design of the study. XW organized the database. XZ performed the statistical analysis. TS and CW wrote the draft of the manuscript. All authors contributed to manuscript revision, read, and approved the submitted version.

## Funding

This work was supported by Agricultural Science and Technology Innovation Project of Shandong Academy of Agricultural Sciences (CXGC2022F05), the central government guides local special projects for science and technology development (YDZX2021101), National Key Research & Development Program of China (China-Israel 2021YFE19800 and 3013005724), Zhejiang Science and Technology Major Program on Agricultural New Variety (2021C02067-7, 2021C02066-5).

## Conflict of interest

The authors declare that the research was conducted in the absence of any commercial or financial relationships that could be construed as a potential conflict of interest.

## Publisher’s note

All claims expressed in this article are solely those of the authors and do not necessarily represent those of their affiliated organizations, or those of the publisher, the editors and the reviewers. Any product that may be evaluated in this article, or claim that may be made by its manufacturer, is not guaranteed or endorsed by the publisher.
